# Evaluation of farnesol orally and topically against experimental cutaneous leishmaniasis: *In -vivo* analysis

**DOI:** 10.1371/journal.pone.0290297

**Published:** 2023-08-28

**Authors:** Harshita Sharma, Rakesh Sehgal, Sanjay Jhacak, Kirti Deshmukh, Ritambhara Nada

**Affiliations:** 1 Department of Medical Parasitology, PGIMER, Chandigarh, India; 2 Department of Natural Products, NIPER, Mohali, India; 3 Department of Histopathology, PGIMER, Chandigarh, India; The University of Burdwan, INDIA

## Abstract

Leishmaniasis is a zoonotic disease transmitted by an obligate intra-macrophage protozoan of the genus *Leishmania* through the infective bite of a vector sandfly. This study investigated the therapeutic efficacy of farnesol, a sesquiterpene compound, for the treatment of cutaneous leishmaniasis (CL) using *in vivo* BALB/c mouse model. In this study, farnesol’s efficacy was compared with the standard drug, paromomycin. It was observed that farnesol significantly reduced lesion sizes and footpad thickness compared to the control group (paromomycin). Lymph node size was also significantly reduced in farnesol-treated mice, indicating its ability to control infection spread. Combination therapy with farnesol and Paromomycin did not demonstrate synergistic effects. These results highlight the potential of farnesol as an alternative therapeutic agent for CL. Further investigations are required to elucidate its mechanism of action and assess potential off-target effects. Optimization of oral delivery methods should be explored to enhance bioavailability. Overall, our findings support farnesol’s efficacy in CL treatment, offering promising prospects for improved disease management.

## 1. Introduction

Leishmaniasis is a zoonotic disease caused by an intracellular protozoan parasite of the genus *Leishmania* that is transmitted by sandfly vectors [1, 2]. It is a disease of great importance for clinical and veterinary health, as it affects various mammalian species, including humans. Leishmaniasis is primarily a disease of low socioeconomic areas, and its occurrence is closely associated with natural disasters, deforestation, infrastructure projects, armed conflicts, and other human activities that lead to the destruction of the vector’s habitat, as well as a weakened immune system and poor living conditions [3]. According to the World Health Organization, leishmaniasis is one of the most neglected tropical diseases, with an estimated 12 million people currently affected, 350 million people at risk, and 0.1 million new cases each year [3–5]. Leishmaniasis presents in six clinical forms, namely cutaneous leishmaniasis (CL), mucocutaneous leishmaniasis (MCL), diffuse cutaneous leishmaniasis (DCL), visceral leishmaniasis (VL), post-kala-azar dermal leishmaniasis (PKDL), and leishmaniasis recidivans (LR) [6]. The clinical presentation of cutaneous leishmaniasis caused by L. aethiopica, L. tropica, and L. major is manifested as ulcers that heal themselves, also known as “oriental sore.” The lesions can be localized or disseminated and generally heal within a few months in immune individuals [7]. *L*. *major*, which is one of the three species that make up the L. tropica complex, is responsible for old world cutaneous leishmaniasis (CL). Since it mainly affects rural areas, it is often referred to as rural zoonotic CL. The disease is primarily reported from the Middle East, India, China, Central Africa, Central and South America, and Central and Western Asia [8]. In India, CL outbreaks have been mostly documented in the arid areas of Rajasthan, Bikaner, and Gujarat, with scattered case reports from Punjab, Assam, and Haryana. However, recently, CL has been reported from other parts of the country, including Himachal Pradesh and Kerala [9–11]. Here is a possibility of local transmission with local vectors and a reservoir, according to reports of the indigenous spread of CL from various parts of India. Although some investigations have highlighted the potential for anthroponotic transmission without the need for an intermediate host, others have indicated the potential for animal reservoirs, such as dogs, wolves, and foxes, to play a role [12]. The infection-transmitting vector species of the subcontinent has been identified as *Phlebotomus papatasi* and *Phlebotomus sergenti*. More than 20 *Leishmania* species, divided into two groups: old world species such as *L*. *major*, *L*. *infantum*, and *L*. *tropica*, and new world species such as *L*. *amazonensis*, *L*. *mexicana*, *L*. *panamensis*, *L*. *braziliensis*, and *L*. *guyanensis*, have been linked to the etiology of the disease. *L*. *tropica* (urban/dry type) and L. major (rural/wet type) are the two most common species from the old world. Old world leishmaniasis is spread by infected female sandflies of the genus Phlebotomus, whereas new world leishmaniasis is spread by infected female sandflies of the genera *Lutzomyia* and *Psychodopygus* [5, 13].

Recently, the Jammu and Kashmir (J&K) union territory (UT) has become a challenging disease focus, with the majority of cases reported from the Chenab Valley, the districts of Poonch and Rajouri in Jammu division, and Kupwara and Baramulla in Kashmir division, which have common borders with Himachal Pradesh and Pakistan, respectively. With its distinction of having hot summers and cold winters, this region’s climate is similar to that of the neighbouring country in many ways, creating a favourable environment for the *Leishmania* species to thrive [3]. There are now concrete efforts by WHO, and the Government of India to control/eliminate Leishmaniasis. Three major strategies are important for this: (1) Prompt diagnosis and treatment of patients as this would decrease the human reservoirs of infections. (2) Use of a good vaccine to prevent the infection and (3) Vector control measures. The decrease/elimination of human reservoirs is important for the control of infection spread. Not only is the present anti-*Leishmania* being used to have serious side effects, but the parasite is also developing resistance to various drugs being used for the treatment. Therefore, we do need safer and better molecules for the treatment of this parasitic infection which would fulfil the goal of eliminating the infection, especially from India.

Although there is currently no vaccination for human usage, there are certain canines, which serve as reservoirs for the parasite. These canine vaccines available for dogs, consisting of fractioned protein components of Leishmania, two such vaccines are commercially available Leishmune and CaniLeish. However, a number of vaccines are through various phases of clinical trials even though none are currently suitable for human application [14]. The current CL treatment strategies include the administration of antimony-based drugs, Glucantime, Paromomycin, and Miltefosine. Paromomycin is also used to treat cutaneous ailments and is currently undergoing a Phase 3 trial for the treatment of new-world human cutaneous leishmaniasis [15]. However, these compounds are associated with a number of adverse effects that limits their usage, like systemic side effects, toxicities, drug resistance, and painful injections which leads to a reduction in patient acceptance. Besides being expensive these are also long and tiring therapies [16, 17]. Patients can suffer damage to their hearts, livers, pancreas, hematopoietic tissues, and renal systems when these compounds fail to provide coverage against *Leishmania*. As a result, it is critical to introduce compounds with fewer complications for CL patients [18]. Compounds with natural antibacterial, anticancer, and anti-inflammatory properties, including anti-leishmanial properties, contribute to the popularity of alternative methods

Naturally occurring compounds and their derivatives are known to possess antimicrobial properties. Farnesol is one such natural compound derived from a range of plants such as citronella, cyclamen, balsam, musk while it is also a constituent of many essential oils [7, 19–21]. Farnesol shows anti-cancer effects on several forms of cancers such as prostate cancer and lung cancer etc. In addition, to being identified as a quorum-sensing molecule of *Candida albicans*, it induces cell death above physiological concentrations which were also observed against bacterial species such as Staphylococcus aureus, *Streptococcus mutans* and the plant pathogenic fungus *Fusarium graminearum* [16, 17]. It has been reported to exhibit significant antimicrobial properties against Plasmodium causing Malaria and Toxoplasma [22]. Farnesol derivatives (trans,trans-farnesol) have also been reported to have a best results highlighting the stereochemistry of the double bond in determining anti-leishmanial activity against *L*. *amazonensis* [23].

In our previous studies, the *in vitro* analysis of farnesol was performed to evaluate IC50 & IC90 values of Farnesol against *L*. *major* promastigotes [24]. The IC50 values of Farnesol were found to be 167.6 ± 4.5 ¼M/ml by MTT assay, while Paromomycin’s were 332.0 ± 5.1 ¼M/ml and the % killing of amastigotes was found to be dose-dependent. Farnesol had an IC50 & IC90 of approx. half that of the standard drug Paromomycin. To gain further insights into the potential mechanisms of action of Farnesol we assessed the *in-silico* drug-binding properties of Farnesol against key enzymes in the ergosterol synthesis pathway. Our findings suggest that Farnesol effectively inhibits Lanosterol 14-demethylase, the terminal enzyme in the pathway. We compared the binding abilities of farnesol and fluconazole against *L*. *major* Lanosterol 14-demethylase and observed that Farnesol exhibited stronger binding affinity than fluconazole. It is worth noting that fluconazole is a well-established inhibitor of Lanosterol 14-demethylase in *L*. *braziliansis*, which causes cutaneous leishmaniasis [25]. However, experimental validation is necessary to confirm the potential of Farnesol as a therapeutic option for this condition hence the need of the present study. We devised the current study to test the efficacy of Farnesol *in-vivo* against an Indian standard strain of *Leishmania major* (MHOM/SU/73/5ASKH) causing Cutaneous Leishmaniasis.

## 2. Material and methods

### 2.1 Drug preparation

Farnesol (Catalog No. F203, Sigma-Aldrich Germany) and Paromomycin (Kwality pharmaceuticals LTD. India) were used for administration in the mentioned animals. Farnesol ointment was prepared for topical application and Paromomycin cream was used. Oral administration was done by dissolving the desired concentration (10, 20, 25 mg/kg) for Farnesol [26] and 50mg/kg for Paromomycin [27] in Carboxymethyl cellulose (CMC). The topical treatment was given twice a day for 10 days and oral treatment was carried out for 20 days twice a day. For the assessment of any combinatory effect of Farnesol and Paromomycin, 250 mM farnesol and Paromomycin cream were applied topically for 10 days twice a day as well.

### 2.2 *Leishmania major* strain

The standard strain *L*. *major* promastigote (strain MHOM/SU/73/5ASKH) [28] was obtained from the lab of Dr. Bhaskar Saha, NCCS, Pune. It was maintained by serial passage *in vitro* culture (passage through BALB/c mice for maintaining virulence). Inbred BALB/c mice, 4–8 weeks old weighing 15 -18gm were used throughout the study. Animals were obtained from IMTECH, Chandigarh, India. Culture was maintained alongside on 10% rabbit Agar plate containing 1X RPMI 1640 medium [29].

### 2.3 Institute animal ethics clearance

The study was approved by the Institute animal ethics committee of the Postgraduate Institute of Medical Education and Research (PGIMER), Chandigarh vide letter no. 831(120^th^/119^th^). Mice were not given anaesthesia at any point in the study. Mice were euthanized by cervical dislocation and then under Sodium penthol I/P (40mg per kg body weight) the carcass were then disinfected by suspending in 1% Sodium hypochlorite solution. The animal was then sent for incineration in yellow-colored sealed envelopes as per the existing guidelines of CPCSEA/ institutional ethics committee.

### 2.4 Parasite inoculation in mice

To initiate *Leishmania* infection, 10 ml of stationary phase culture was resuspended in 1ml of PBS/normal saline to prepare an inoculum of 50μl, 10^6^−10^7^ stationary phase promastigotes injected intradermally in each hindfoot of BALB/c males with the help of 1ml syringe (purchased from Dispovan).

### 2.5 Groups of mice

**Fig 1:**
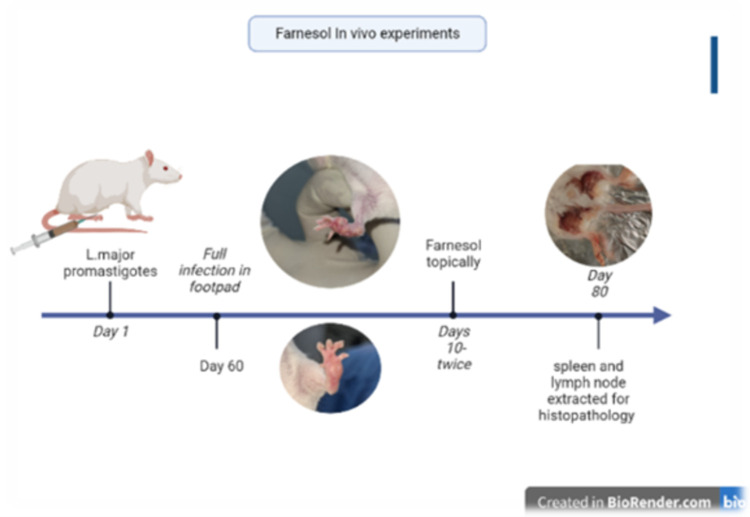
*In vivo* experimental workflow (created with BioRender.com).

The mice were then distributed into the following groups: the test group which received Paromomycin (gold standard) and Farnesol and the control group which received the ointment void of drugs. These groups were further divided into two groups—oral and topical (Table 1). Treatment was initiated upon the development of nodules and ulcers at the peak of infection i.e., 2 months post-infection. The infected mice were treated by applying the preparations twice daily at the lesion site (early morning and late afternoon) in BALB/c mice a total of 5 mice weighing 15–16 kg and 4–8 weeks old were set up in each experimental group and treatment was given two-months post-infection for a period of 10 days topically and 20 days orally. The selection of the doses for Farnesol [26] and Paromomycin [27] were adopted from previous studies. The workflow of the *in vivo* experiments is described in Fig 1.

**Table 1:** Farnesol topical- Number of mice in each group for efficacy studies.

Group I			Group II		
**Farnesol concentration(mg)**		**No. of Mice Oral group**	**Farnesol concentration(mM)**		**No. of Mice Topical group**
		**60 Days** **5** **5** **5** **5** **5** **5**			**60 Days** **5** **5** **5** **5** **5** **5**
**Negative control**			**Negative control**		
**Positive control**			**Positive control**		
**IC 50**	**15 mg/kg**		**IC 50**	**500**	
	**20 mg/kg**			**250**	
	**25 mg/kg**			**100**	
	**Paromomycin 50 mg/kg**			**Paromomycin cream**	

### 2.6 Parameters for drug toxicity

The Parameters for toxicity evaluation in mice were as follows: (1) Chemo-suppression- In every tested group, the average mortality “mice” and “mean survival time” (MST) will be determined arithmetically [30]. (2) Determination of Body weight change during the study-The mice will be kept for following before infection (0 days) and last infection (28 days) including the dose regimen to determine the "mean survival time " (MST) of mice in every group [31]. (3) Mean Body Weight = Mean body weight of mice in a group/Total no of mice in that group. (4) Histopathology- Histopathology was done by the processing of the sections of vital organs as lymph node, spleen by specimen accessioning, gross examination, tissue fixation, tissue processing (dehydration, clearing, impregnation) tissue embedding, tissue sectioning and then slide staining by Hematoxylin & Eosin [32]. (5) Lymph node size and weight- Lymph node size and weight was measured in infected mice and was compared with healthy mice [33]. (6) Parasite load- Parasites were counted by LD index in stamp smear- counting amastigotes in 1000 nucleated cells × organ weight.

### 2.7 Preparation of Farnesol-based ointment

The Procedure for making Ointment for Different Concentrations of Farnesol as per the following formulations: 0.5g of hard paraffin pellets were heated at 95°C in a water bath. On melting of the paraffin pellets, a transparent liquid was observed to which cetosteryl alcohol (0.5 g) was added. Wool fat/Lanolin (0.5 g) was added and heated till a transparent liquid was observed and then farnesol @ 4M, 500 mM, 250 mM & 100 mM respectively was added. Till the evaporation of ethanol (transparent liquid again), this mixture was boiled and stirred. White paraffin wax was added to this transparent mixture and heated for another 2 minutes. Now the mixture was stirred continuously at room temperature till a semi-solid ointment was formed (Table 2). The white soft paraffin served as a greasy ointment base to incorporate the above- mentioned materials. Lanolin was added to enhance the hydrophilicity of the preparations. No preservative was added and the ointments were kept at 4°C.

**Table 2:** Contents required for farnesol ointment.

Material Required	Ointment (10 ml)	Concentration of Farnesol in ointment	Preparation of Farnesol Concentration(10ml)	
**Wool Fat**	**0.5 g**	**2M**	**Drug**	**Ethanol**
			**5 ml**	**5 ml**
**Hard Paraffin**	**0.5 g**	**500 mM**	**1.25 ml**	**8.75 ml**
**Cetosteryl alcohol**	**0.5 g**	**250 mM**	**625 ¼l**	**9.4 ml**
**White Soft Paraffin**	**8.5 g**	**100 mM**	**250 ¼l**	**9.7 ml**

#### 2.7.1 Characterization studies of ointment

Physical appearance and physicochemical properties were evaluated for farnesol ointment. The physical appearance of the ointment was characterized by colour, odour, and texture. Physicochemical properties such as (a) Viscosity- The viscosity of the formulations was determined using a Brooke field viscometer at 272°C and a spinning speed of 100 rpm; (b) Spreadability- Two sets of rectangular glass plates (diameter 15 cm) were taken. Farnesol ointment (1 g) was placed over one of the plates and the other plate was placed on top of the ointment. The standard weight (125 g) was applied on the upper plate for 1 minute. The spreadability of the ointment was determined by measuring the area of the circle formed after the spreading of the ointment; (c) Stability Studies-Accelerated stability studies were performed on formulations of different concentrations of farnesol for a period of 2 months. Stability tests were performed considering storage conditions of temperature and humidity i.e., 40 ± 2°C and 75 ± 5% respectively; and, (d) pH analysis-The pH of the ointment was evaluated using a digital pH meter.

#### 2.7.2 GC-MS (Gas-Chromatography-mass spectroscopy) analysis conditions for farnesol ointment

GC-MS analysis was done to confirm the presence of Farnesol in the ointments at different concentrations, in EI +ve mode by using Perkin Elmer Clarus 600 Gas chromatograph and Perkin Elmer Clarus 600 C mass spectrometer using Elite-5MS (30×0.25) column. A temperature program comprising an injector temperature of 220°C was used. The initial oven temperature was 120°C which was held for 2 minutes. The temperature of the oven was increased up to 230°C by raising the temperature by 10°C every minute. The carrier gas used was helium (1.0ml/min). 0.5 ¼l sample was injected in slit mode. Farnesol ointment samples were prepared for GCMS analysis. A standard farnesol sample was prepared by dissolving it in hexane. For the preparation of the ointment of the farnesol sample, a solvent extraction method was used using various solvents in the proper ratio of the mixture. The solvent used was petroleum ether (40%), chloroform (40%), methanol (15%), and *n*-butanol (5%). Samples were subjected to centrifugation for 30 min at 8000 rpm. To prepare the sample for GCMS, the centrifuge obtained was dissolved in a hexane [34].

### 2.8 PCR of lymph node and spleen of treatment groups

**Controls.** For pathogen controls, DNA from culture isolates of *L*. *major* were used as applicable. Nuclease free water was also used in all reactions.

#### Parasitic nucleic acid extraction

DNA extraction from lymph node and spleen samples, was done using QIAamp DNA mini kit (Qiagen GmbH, Hilden, Germany) according to manufacturer’s instructions with minor modifications in the protocol: 180¼l of ATL (tissue lysis buffer), 20μl Qiagen proteinase K used to digest proteins was added to 200μl of tissue sample and homogenized followed by incubation at 56°C for 2 hours with vortexing after every 30 minutes. This was followed by addition of 200 μl of lysis buffer (AL) with vortexing and incubation done at 65°C for 30 min. After this, 200μl of ethanol (96–100%) was added and mixed by vortexing for 30 sec followed by brief centrifugation. The mixer was applied into the QIAamp Spin Columns and centrifugation was done at 8000 rpm for 1 min. The supernatant was discarded and centrifugated at 8000 rpm after addition of 500μl of wash buffer (AW1) for 1 min. This was followed by addition of 500μl wash buffer (AW2) and centrifuged at 14000rpm for 3 minutes. The final step involved elution of the DNA by adding 40μl of elution buffer in the centre of the column fixed to a fresh 1.5 ml eppendorf tube followed by incubation at room temperature for 5 min and centrifugation at 10000 rpm. The extracted DNA was then preserved at -20°C until further processing.

#### Detection of parasites by conventional PCR

Conventional PCR was performed in all the samples for kDNA minicircle [35] gene of *L*. *major* and for housekeeping *18s* gene universal primers [36, 37] (refer to Table 3). The total volume of the reaction mix was 25¼l with 1x PCR buffer, 1¼M of dNTPs and 10¼M of *L*. *major-specific* primer was added to 2¼l of the DNA sample with an optimum volume of Taq DNA polymerase (5 units). Amplification condition was 10 min at 94°C, proceeded by 34 cycles of 1min at 94°C, 1:30 min at 56°C and 1:30 min at 72°C with a final extension at 72°C for 10 mins. PCR products were run on 1.5% of agarose gel.

**Table 3:** *L.major*- specific primers sequence as follow.

Lm F 5′- GGG GTT GGT GTA AAA TAG GCC -3’
Lm R 5′- CTA GTT TCC CGC CTCCGA G -3’ [35]
**18s F 5′- GAGAAACGGCTACCACATCC -3’**
**18s R 5′- GGACACTCAGCTAAGAGCATCG -3’**

### 2.9 Combination therapy

A pilot study was conducted to evaluate any enhanced effects of combination therapy on *L*. *major* induced cutaneous lesions. The best dose of Farnesol was applied topically on footpad in combination with Paromomycin cream [27]. Lesion size of infected mice was compared with healthy mice with the help of vernier calipers [33]. Four groups each containing 5 mice were set up randomly with farnesol at 250 mM/ml, paromomycin, farnesol + paromomycin, positive and negative controls respectively (Table 4).

**Table 4:** Combination therapy: Groups of paromomycin and farnesol topically.

Groups	No. of mice (Topical)
**Negative control**	5
**Paromomycin alone**	5
**Farnesol alone**	5
**Paromomycin + Farnesol**	5

### 2.10 Statistical analysis

One-way ANOVA were used to analyze mean values. All experiments were performed in duplicates or triplicates. The experimental data were summarized using mean ± SEM. The statistically significant level for differences between mean values was accepted at P < 0.05. Graph pad 9.3.1 was employed for statistical analyses.

## 3. Results

### 3.1 GC-MS analysis of farnesol ointments

**Fig 2:**
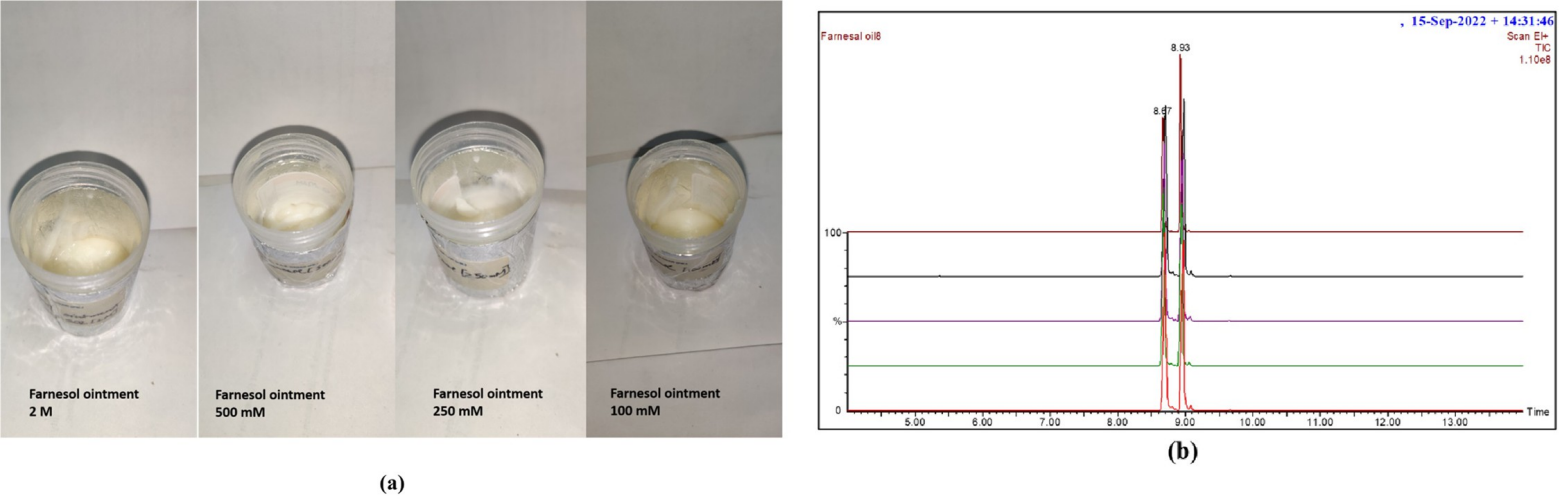
GC-MS chromatograms for ointment samples and farnesol (E/Z) **(a)** Farnesol ointments at different concentrations; **(b)** GC-MS chromatograms of farnesol (E/Z) and samples merged.

The results from GC-MS analysis of farnesol ointments at different concentrations displayed peaks which show the absence of hydroxyl group in GC-MS which is originally displayed in the standard oil sample of farnesol’s GCMS analysis as in its chemical formulae. Moreover, all sample of different concentrations of farnesol had peaks as found in the standard of farnesol which confirms the presence of drug in all ointments (Fig 2). GC-MS analysis is tabulated below for farnesol Entgeggen (E) and Zeusamann (Z) isomers (Table 5).

**Table 5:** Peak values for chromatograph of farnesol (E/Z) and ointment samples.

Concentrations	% Area	Retention time	Height of peak	(m/z)	Molecular formula
Farnesol Stn^d^	46.24	8.66	70,8941.52	223	C15H26O
	53.27	8.93	109,5156.56	223	C15H26O
**2M**	49.49	4.7	784,876.864	204	C15H25
	48.46	4.9	750,743.360	204	C15H25
**100 mM**	50.47	8.6	676,189.248	204	C15H25
	48.12	8.95	614,861.760	204	C15H25
**250 mM**	49.71	8.69	736,016.320	204	C15H25
	48.22	8.97	692,632.640	204	C15H25
**500 mM**	49.06	8.71	744,941.440	204	C15H25
	48.86	8.98	773,433.856	204	C15H25

### The results obtained from characterization studies were as follows

#### (a) Physical appearance

The Ointment was white in color with fruity fragrance and smooth texture.

#### (b) Physicochemical properties

**Fig 3:**
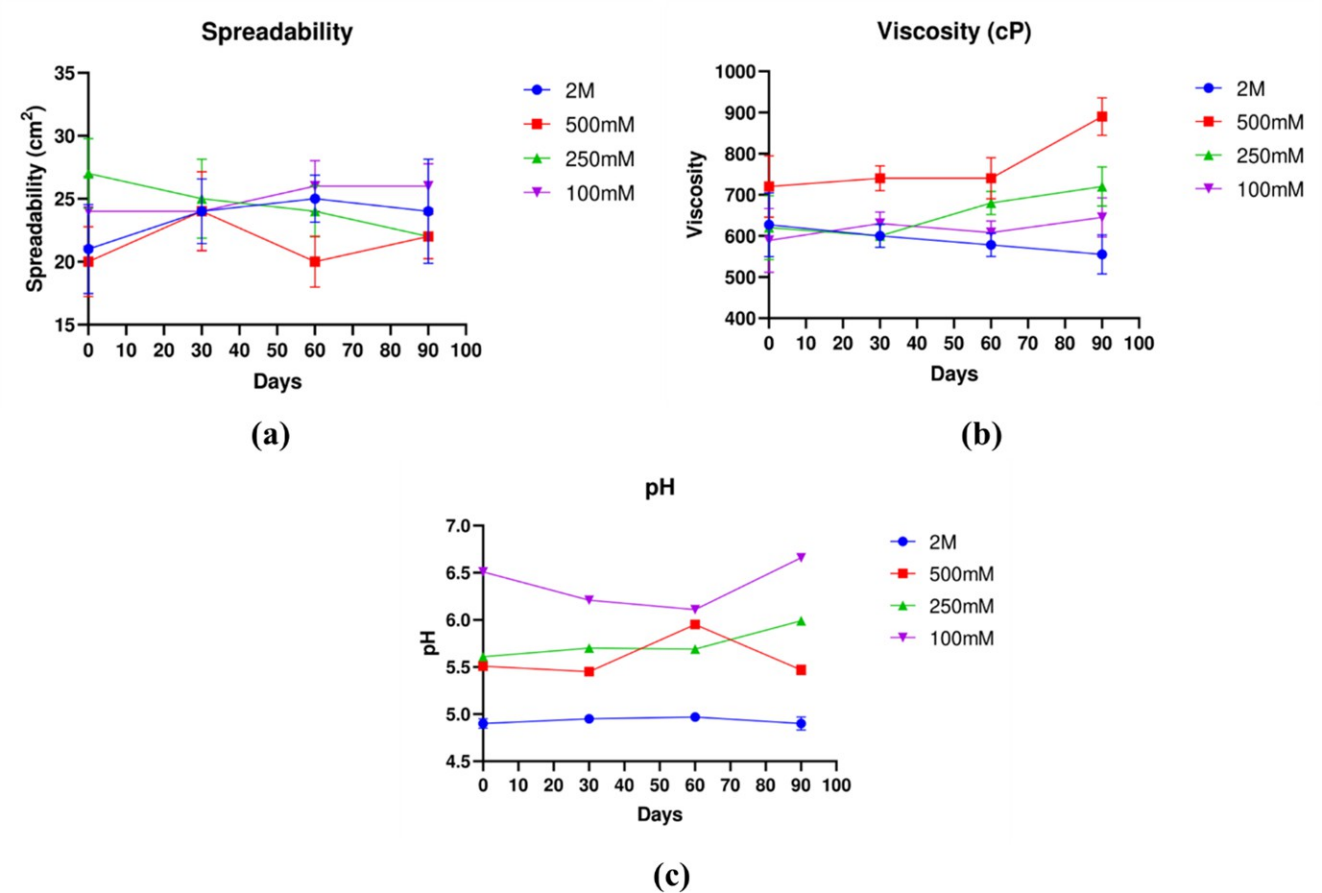
Characterization studies of farnesol **a.** pH; **b.** Spreadability; **c.** Viscosity.

Physicochemical properties pH was in the range of 5–6.5 which is not harmful for the growth of *Leishmania* parasites as they grow in the same pH range; spreadability was 20–30 cm ^2^ which means it can be spread on a large area of the wound easily, and viscosity for all samples was 600–700 Pa which falls in the acceptable range for drugs used for dermal application (Fig 3).

### 3.2 Infection model validation by PCR

**Fig 4:**
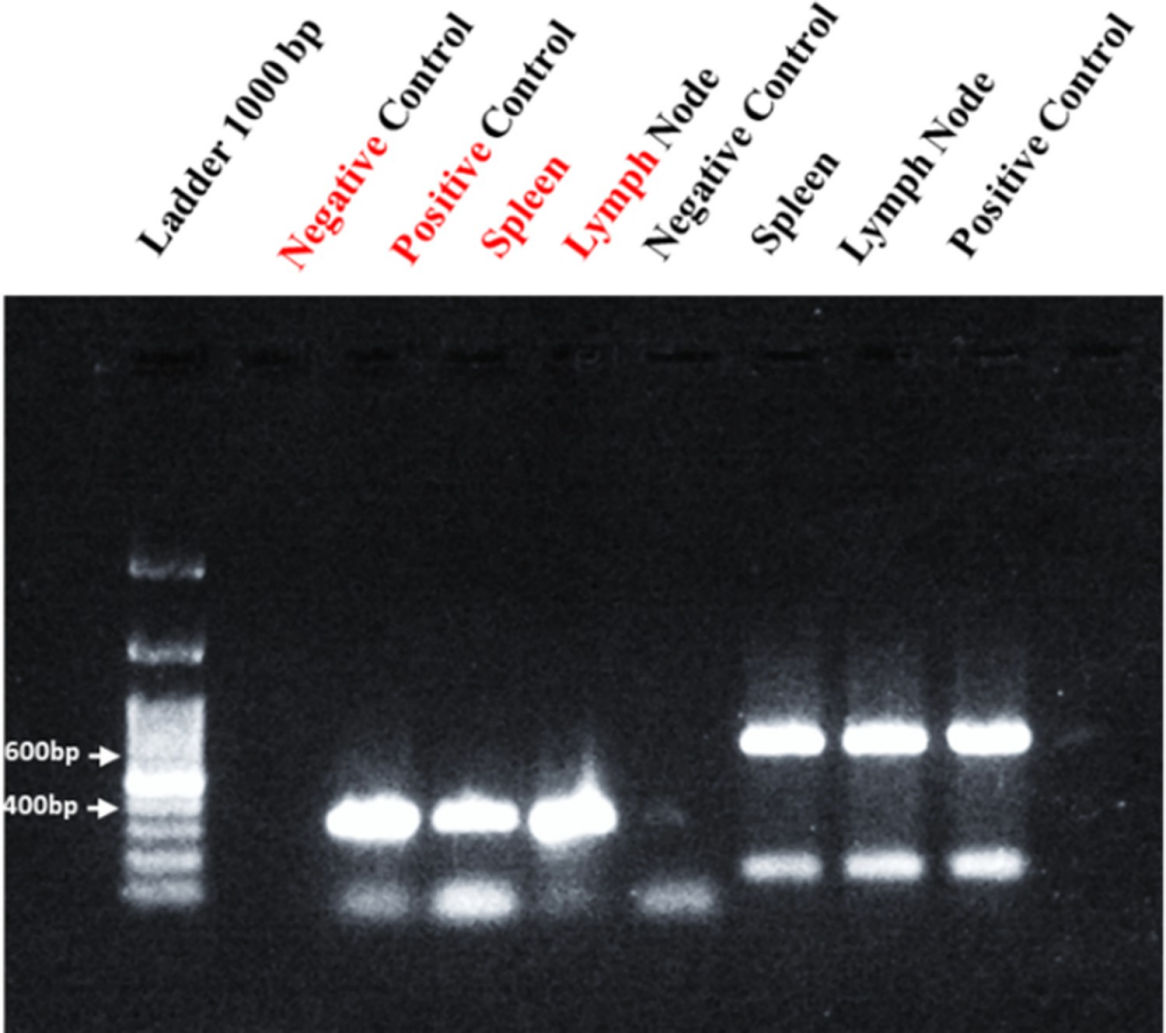
Gel Electrophoresis images with bands at 390 bp for 18s gene & 650 bp for *Leishmania major* recovered from the spleen and lymph node of infected mice.

The samples of popliteal lymph nodes and spleen obtained from the treated, negative and positive groups of mice were analyzed by PCR where positive control was taken from *in vitro* maintained culture to confirm the development of an infection model of *Leishmania major and* with *18s* housekeeping gene. Lymph node and Spleen were found positive by Conventional PCR which confirm the established of an infection model of *L*. *major* for further toxicity analysis. All results were carried out in duplicates (Fig 4).

### 3.3 Parameters for *in vivo* toxicity analysis

#### 3.3.1 No significant change in body weight of mice

**Fig 5:**
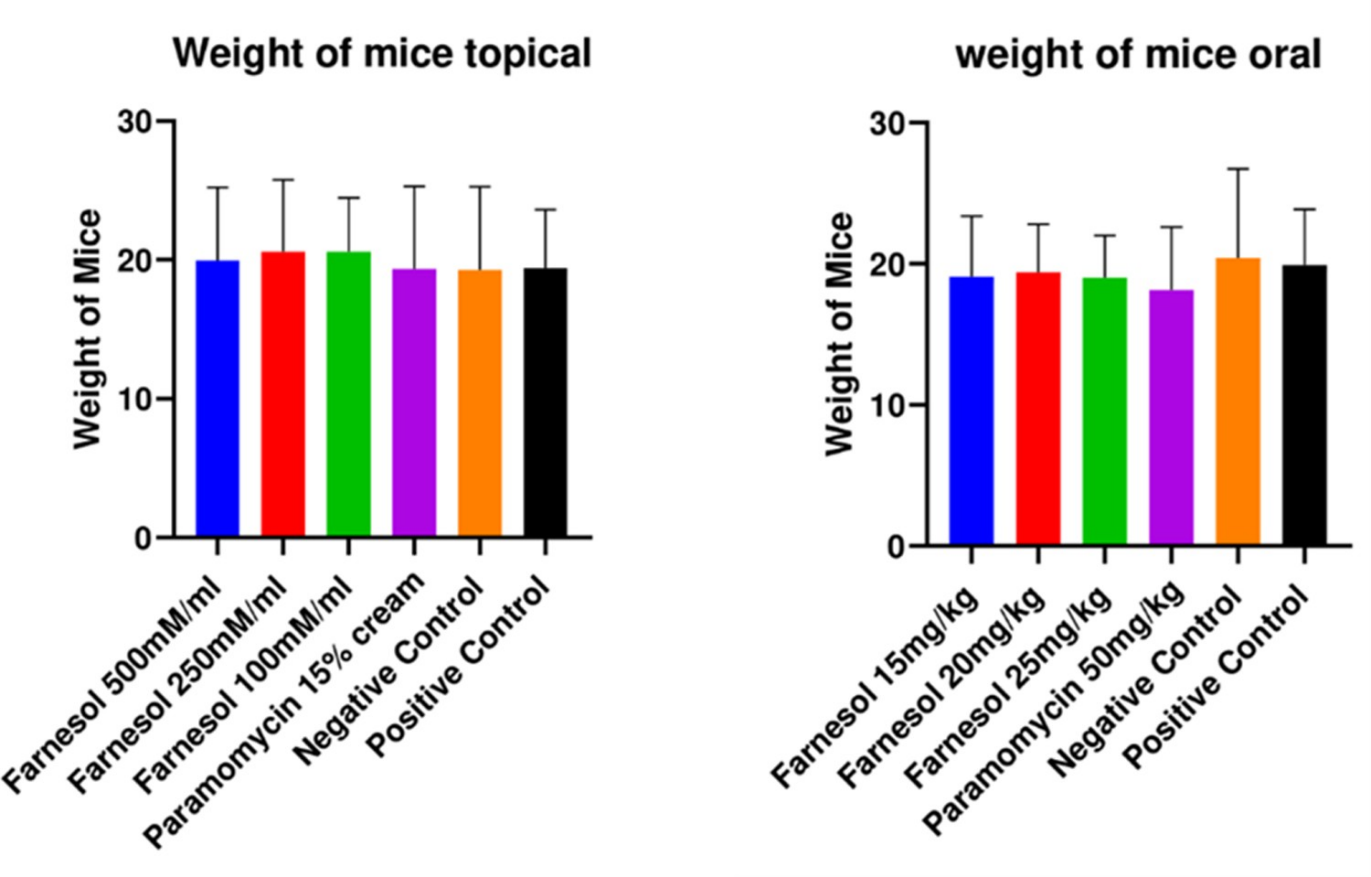
Body weight change of mice topical & oral group for the duration of the study.

Our results showed that farnesol or Paromomycin had no significant decrease in mice weight throughout the study. There was no weight change even in the positive controls which shows that decrease in weight had no association with cutaneous infection caused by the current strain used in the study. There were no significant association of infection to health of mice apart from temperature increase observed on holding the mice and lethargy at the peak of infection i.e., 2 months post infection (Fig 5).

#### 3.3.2 Change in size and weight of Lymph node and spleen

**Fig 6:**
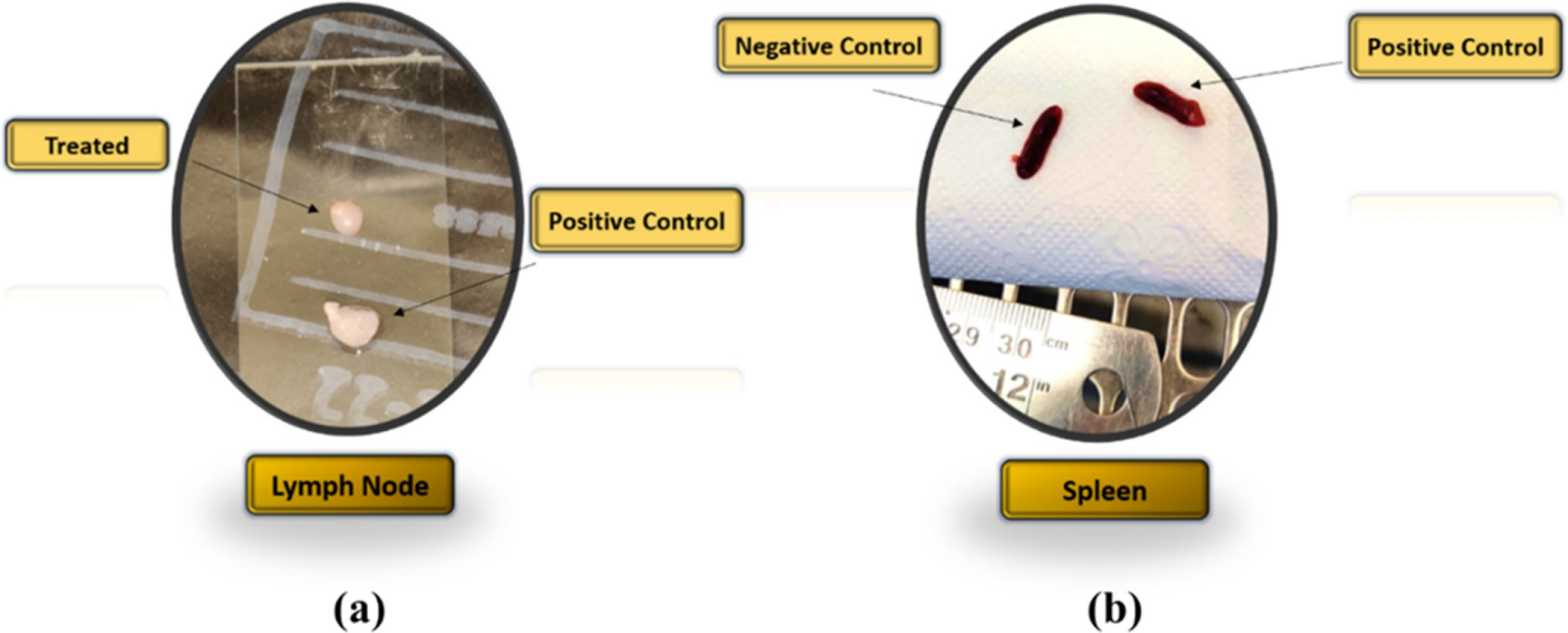
Comparison of Lymph node and spleen size in treated groups, Positive and Negative control groups **(a)** Popliteal lymph node **(b)** Spleen.

The change in size of popliteal lymph node and spleen was observed among treated and non-treated groups of drugs. The size of lymph node was significantly reduced in treated groups when compared to the lymph node of non-treated groups of mice (Fig 6A), but there was no change in the size of spleen in treated groups when compared to the non-treated groups of mice (Fig 6B).

**Fig 7:**
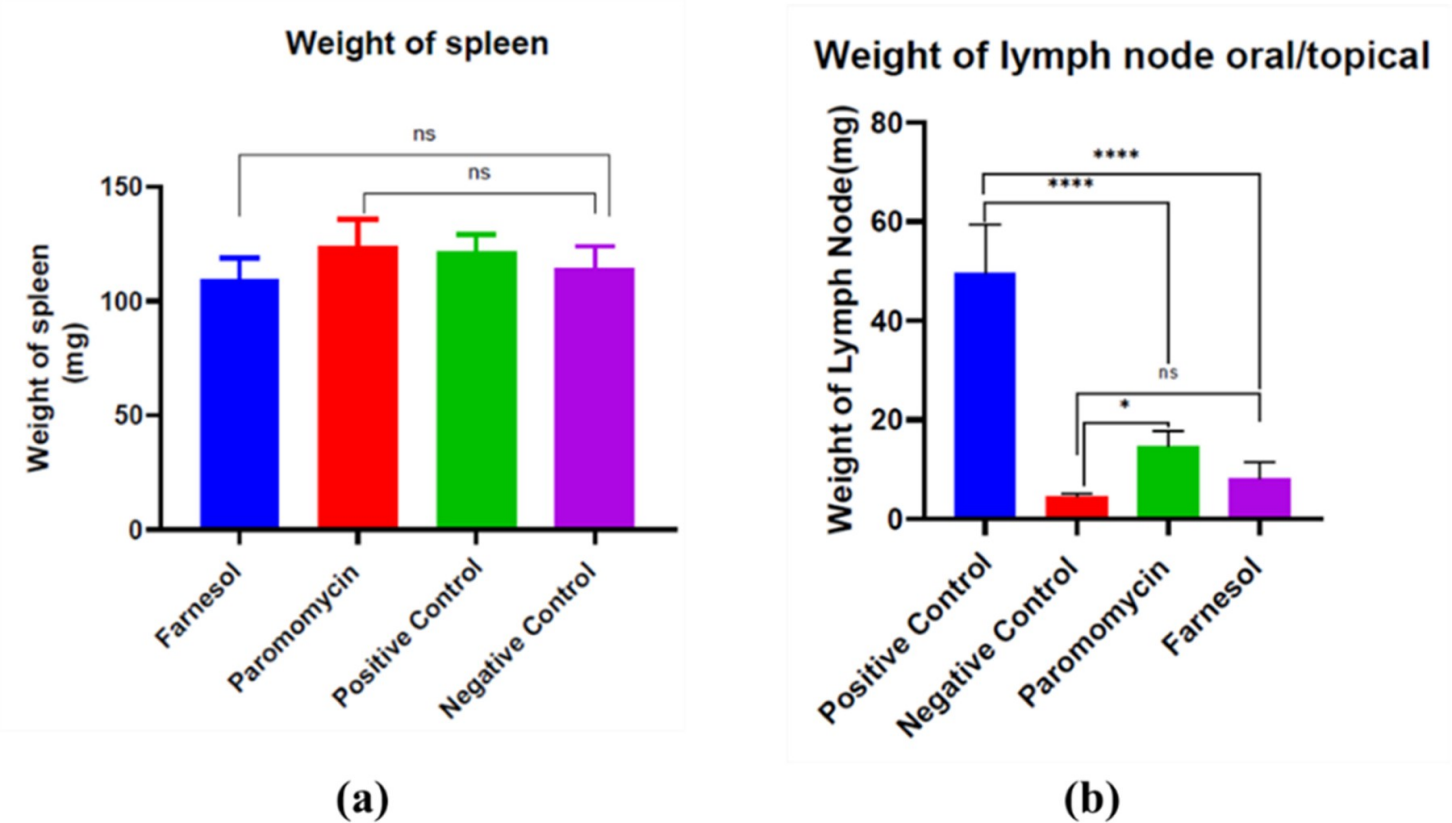
Comparison of Lymph node and spleen weight in treated groups, Positive and Negative control groups **(a)** Spleen **(b)** Popliteal lymph node.

Weight change of popliteal lymph node and spleen were measured as a parameter of drug efficacy. The weight of lymph node for farnesol treated mice was 8.26 ± 2.29 mg/kg while that of Paromomycin was 14.6 ± 3.3 mg/kg compared to treated 49.6 ± 10.4 mg/kg and that of non-treated groups was 4.46 ± 0.4 mg/kg was significantly lower for farnesol treated groups in comparison to Paromomycin both for topical and orally treated groups of mice (Fig 7B). The weight of spleen of farnesol treated group was 109.44 ± 7.7 mg/kg, Paromomycin treated was 124.1 ± 13.2 mg/kg, positive control was 124.8 ± 8.3 mg/kg, and that of negative control was 119.54 ± 8.52 mg/kg. The treated groups exhibited a significant reduction in size and weight of the popliteal lymph node compared to the non-treated groups, indicating the efficacy of farnesol and Paromomycin, while no significant changes were observed in the size and weight of the spleen, suggesting the limited impact of the treatments on spleen parameters (Fig 7A).

#### 3.3.3 Significant change in lesion size/ footpad thickness

**Fig 8:**
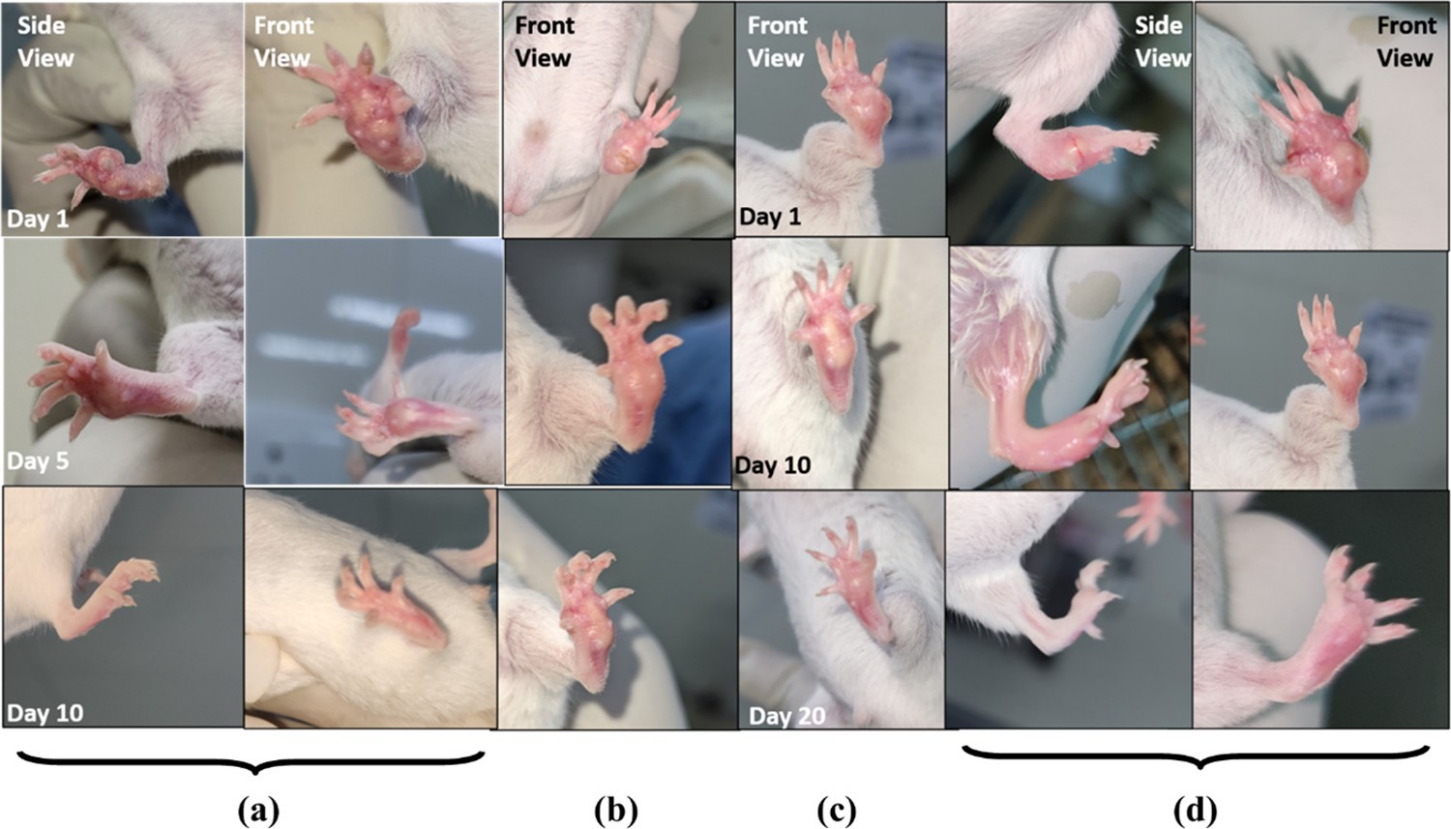
Farnesol topical and oral therapy in comparison to standard drug paromomycin **(a)** Farnesol topical **(b)** Paromomycin topical **(c)** Paromomycin oral **(d)** Farnesol oral.

*a*. *Qualitative reduction in Footpad thickness*. In the 10-day treatment period of topical and orally treated mice, the footpad thickness was measured at Day 1, Day 5 and Day 10 of treatment. The results from qualitative analysis of oral and topically treated groups of farnesol demonstrated an appreciable reduction in lesions developed in footpad of mice compared to the control groups. There was a significant decrease in the footpad thickness of farnesol treated mice at 100–250 mM/ml than that of the standard drug Paromomycin (Fig 8). The lesions were healed completely in groups treated with farnesol at 250 mM/ml while paromomycin treated lesions still had some swelling, as shown in Fig 8 after treatment for a period of 10 days/twice, same as the test drug. The findings reveal that both topical and oral administration of farnesol result in a substantial reduction in footpad lesions and significant decrease in footpad thickness, surpassing the effectiveness of the control groups and outperforming the standard drug Paromomycin.

**Fig 9:**
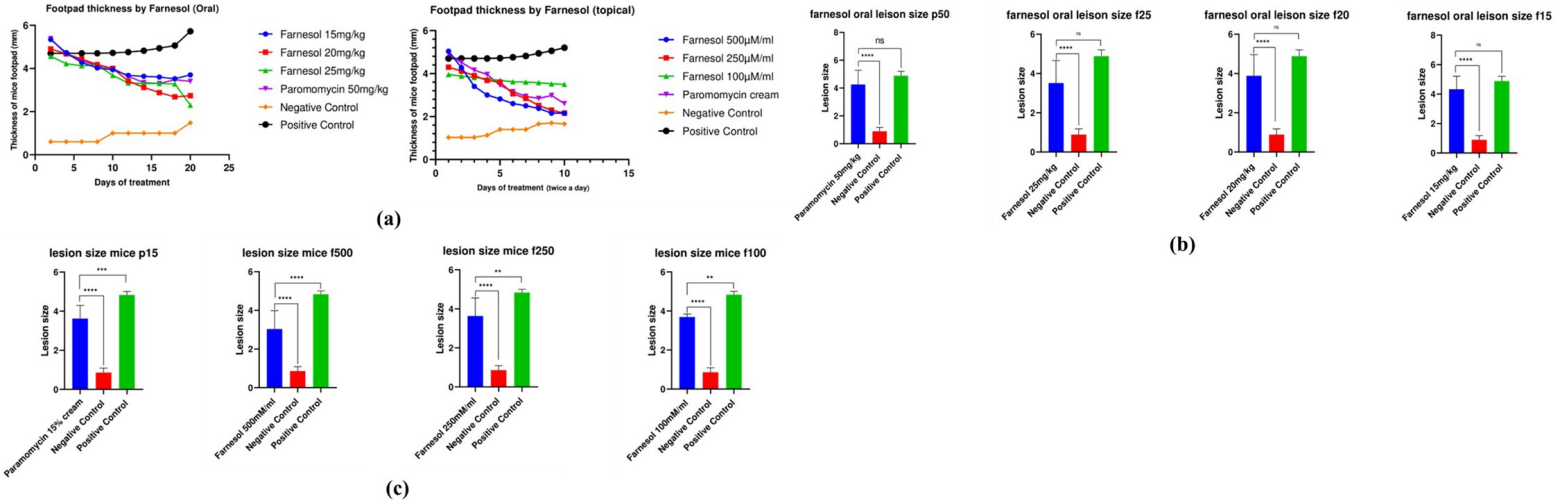
Footpad thickness **a.** Footpad thickness oral and topical curves **b.** Statistical analysis of Topical group; **c.** Oral group.

*b*. *Quantitative reduction of footpad thickness*. The footpad thickness was measured with the help of vernier caliper at Day 1, 5 and 10 of the treatment for Topical application and 1, 10 and 20 for Oral treatment. Footpad thickness was 5.2 mm for positive control while it was reduced to 2.16 mm in case of Farnesol treated mice at 250 mM/ml and 25mg/kg, both in topical and oral groups respectively while that of paromomycin at 50mg/kg was 3.16 mm. Farnesol treated footpad of mice were healed completely by Oral-25mg/kg and Topical treatment at 250mg/kg in comparison to paromomycin at 50mg/kg. Statistical analysis for both oral and topical groups are shown in Fig 9 for both the drugs in comparison with positive and negative controls respectively. Farnesol treatment, administered topically and orally, exhibits a significant reduction in footpad thickness compared to paromomycin treatment, resulting in complete healing of the footpad in mice.

#### 3.3.4 Reduction of parasite load

**Fig 10:**
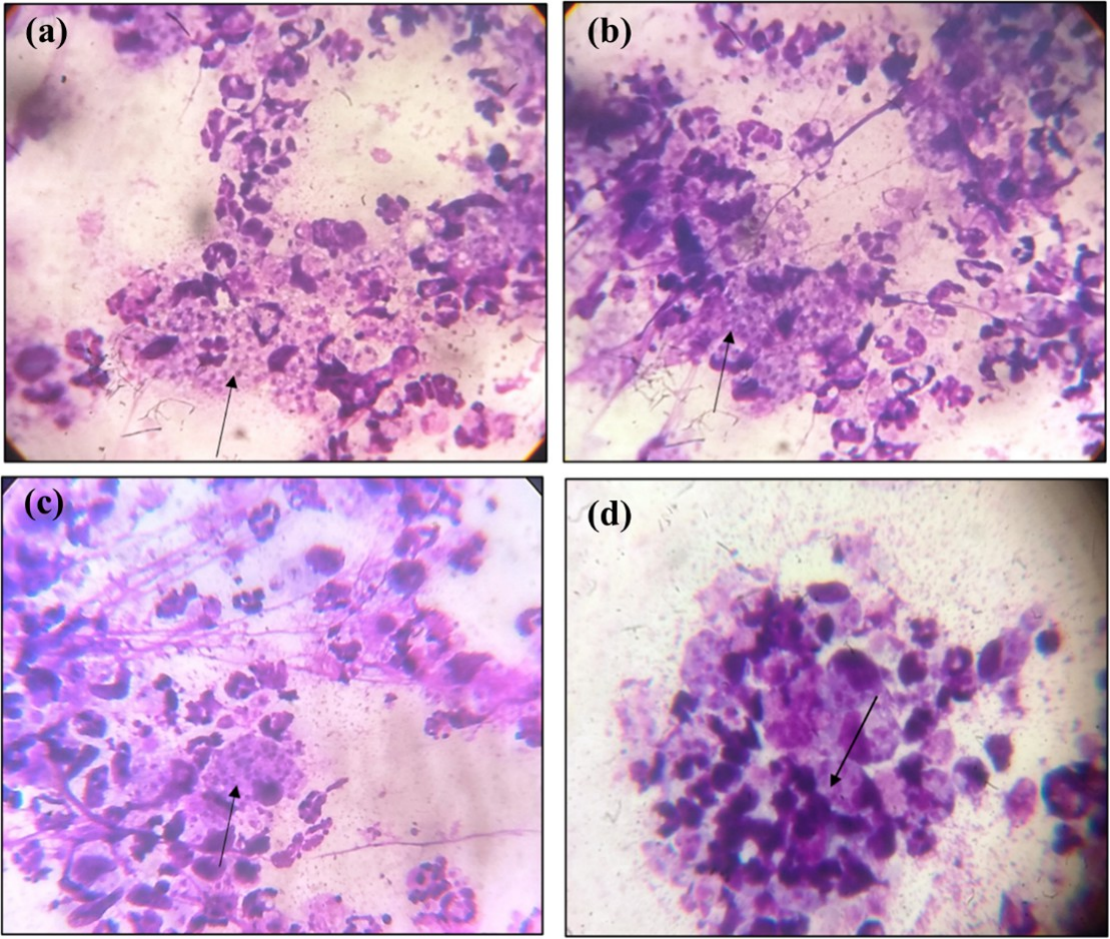
Lymph Node stamp smear for parasite load analysis by LD index **a.** Positive control; **b.** Farnesol oral; **c.** Farnesol topical; **d.** Paromomycin.

For determining the parasite load in the lymph node Leishman Donovan index was calculated by preparation of stamp smears of popliteal lymph node (Fig 10) of all groups of mice in duplicates. The formula for calculation of LD Index was as follows:

**No. amastigotes in 1000 nucleated cells of lymph node × Weight of lymph node (Leishman Donovan unit/Index)**

**Fig 11:**
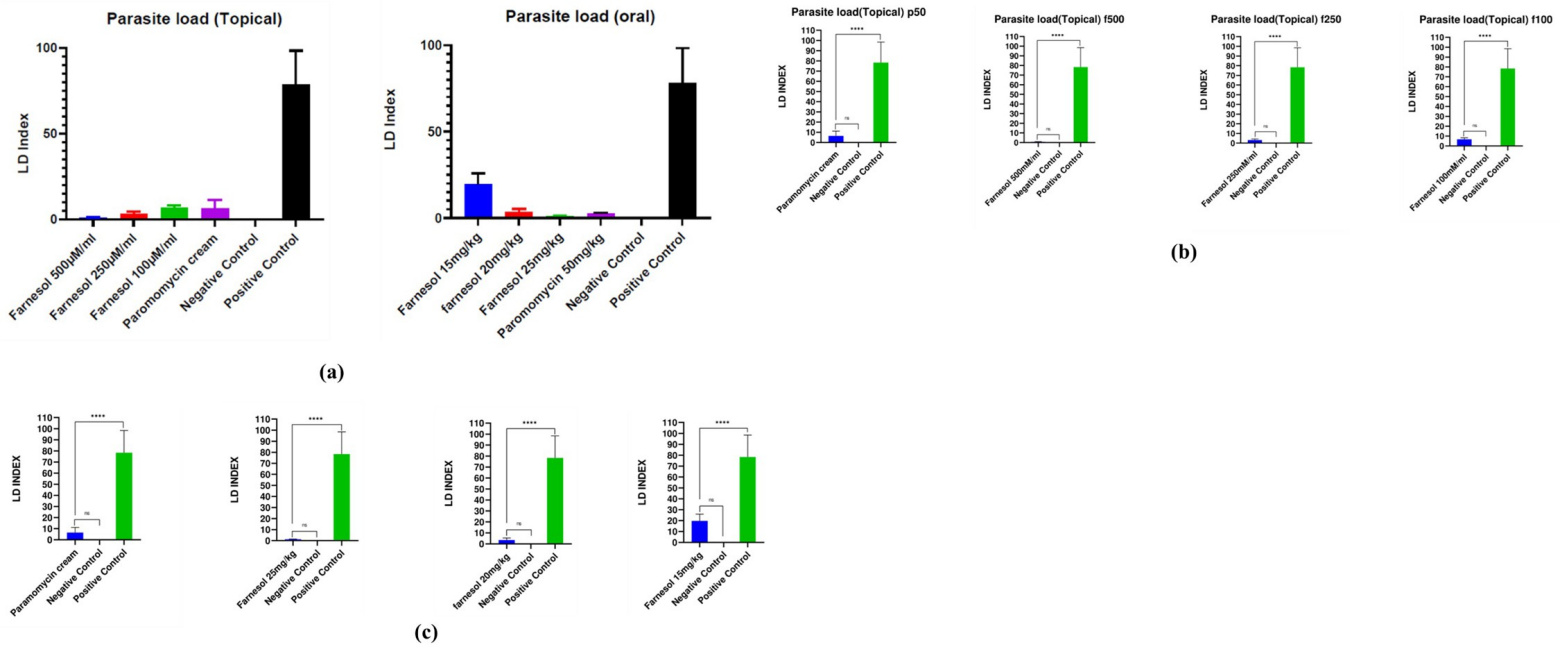
**a.** Parasite load topical & oral curves **b.** Statistical analysis of Topical group**; c.** Oral group.

Parasite Load was reduced significantly in both topical and oral groups of Farnesol in comparison to paromomycin treated groups and positive controls. While comparatively there was more reduction of parasite load in orally treated groups compared to topical groups of mice. The LD index of the topical group of farnesol at 250 mM was 2.22 compared to Paromomycin cream with an LD index of 13.13 and which is significantly very less than the positive control with an LD index of 82.77 for topically treated mice (Table 6). The LD Index was 1.2 for 25mg/kg of farnesol compared to 78.34 LD for positive control of orally treated groups (Table 6). In the groups of Farnesol, orally treated mice had a lower parasite load compared to topically treated groups as shown in Table 7. Statistical significance of all the test groups are shown in Fig 11 along with positive and negative control groups.

Farnesol treatment, both orally and topically, significantly reduces parasite load in comparison to paromomycin treatment and positive controls, with greater reduction observed in orally treated groups.

**Table 6:** LD Index of Topically treated mice.

Groups (Oral Treatment) (n = 3)	LD Index (Mean ±SD)
**25 mg/kg**	**1.2 ± 12.2**
**20 mg/kg**	**5.42 ± 5.3**
**15 mg/kg**	**13.21 ± 8.52**
**Paromomycin 50 mg/kg**	**3.48 ± 2.68**
**PC**	**78.34 ± 20**

**Table 7:** LD index of orally treated mice.

Groups (Topical application) (n = 3)	LD Index (Mean ±SD)
**500 mM**	**2.22 ± 2.71**
**250 mM**	**9.195 ± 5.27**
**100 mM**	**11.71 ± 7.8**
**Paromomycin Cream**	**13.13 ± 9.28**
**PC**	**82.77 ± 20.38**

### 3.3.5 Histopathological analysis confirms inhibitory effects of farnesol on lymphatic cells

**Fig 12:**
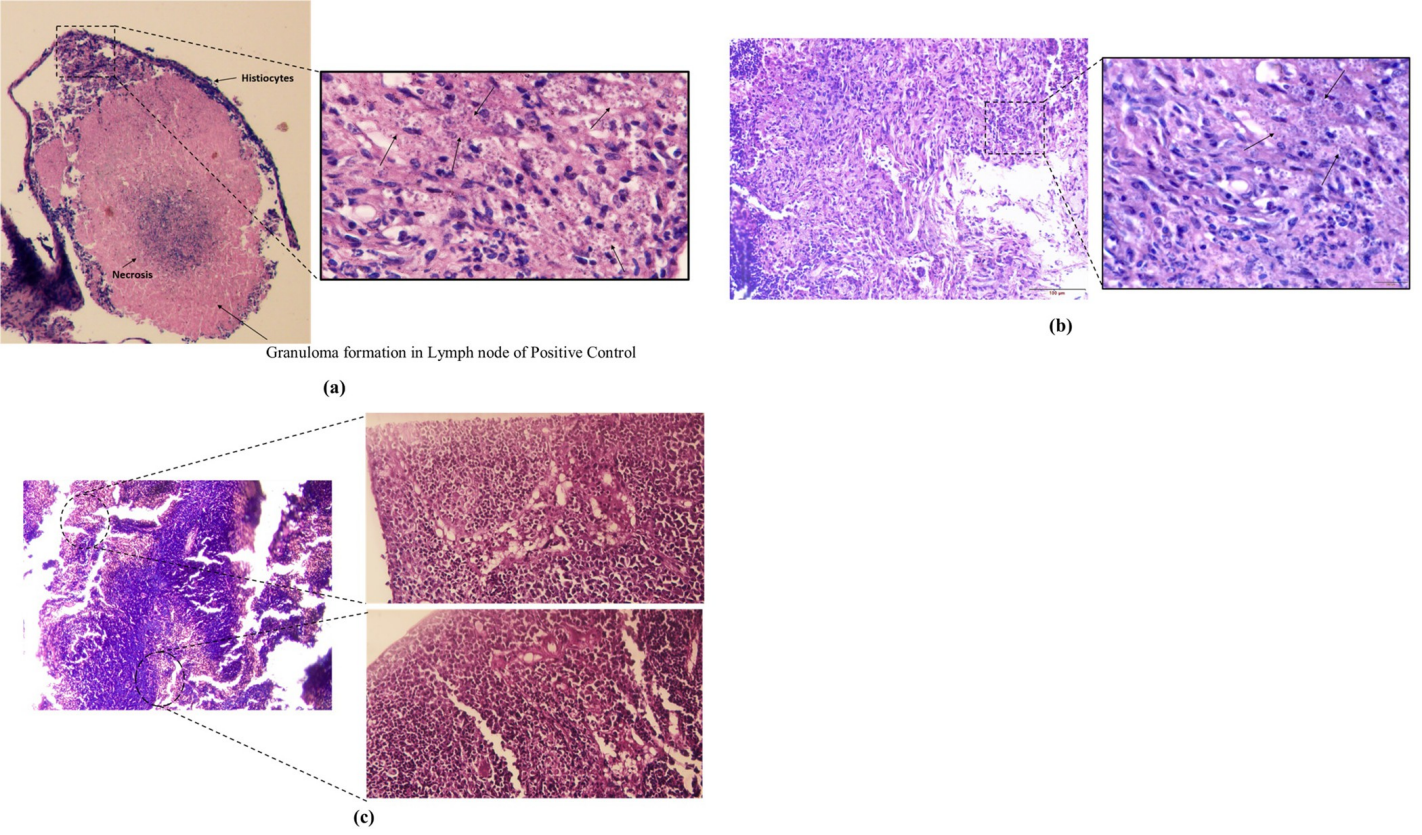
Histopathology analysis of Lymph Node **a.** Positive control; **b.** Farnesol treated group; **c.** Negative control.

**Fig 13:**
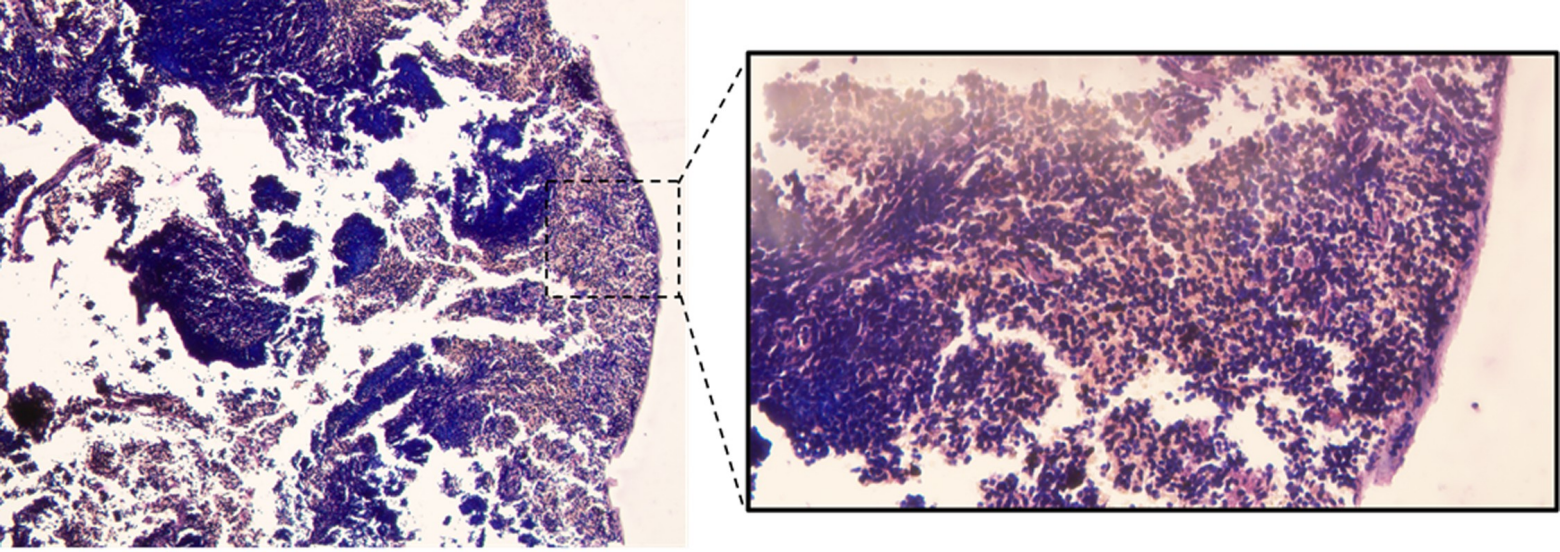
Histopathology analysis of spleen positive control.

For histopathological analysis, 3 mice from each group were sacrificed after the completion of treatment. From the histopathological slides of untreated groups, it can be clearly seen to contain a large migration of histiocytes. There was granuloma formation and necrosis of cells with macrophages containing a large burden of amastigotes phagocytized within. While the treated groups showed Histiocytes/macrophages migrating to lymph nodes of oral and topical groups of both paromomycin and Farnesol treated groups, there was no granuloma formation observed. LD bodies were present but parasite load was much reduced in Farnesol treated groups in comparison to non-treated groups (Fig 12A–12C). No LD bodies or macrophages migrated to spleen in the positive control compared to negative control which now confirms no infection in Spleen (Fig 13). Treatment with Farnesol reduces parasite load and inhibits granuloma formation in histopathological analysis, indicating its potential efficacy in combating the infection.

### 3.3.6 Antagonistic effect in combination therapy

**Fig 14:**
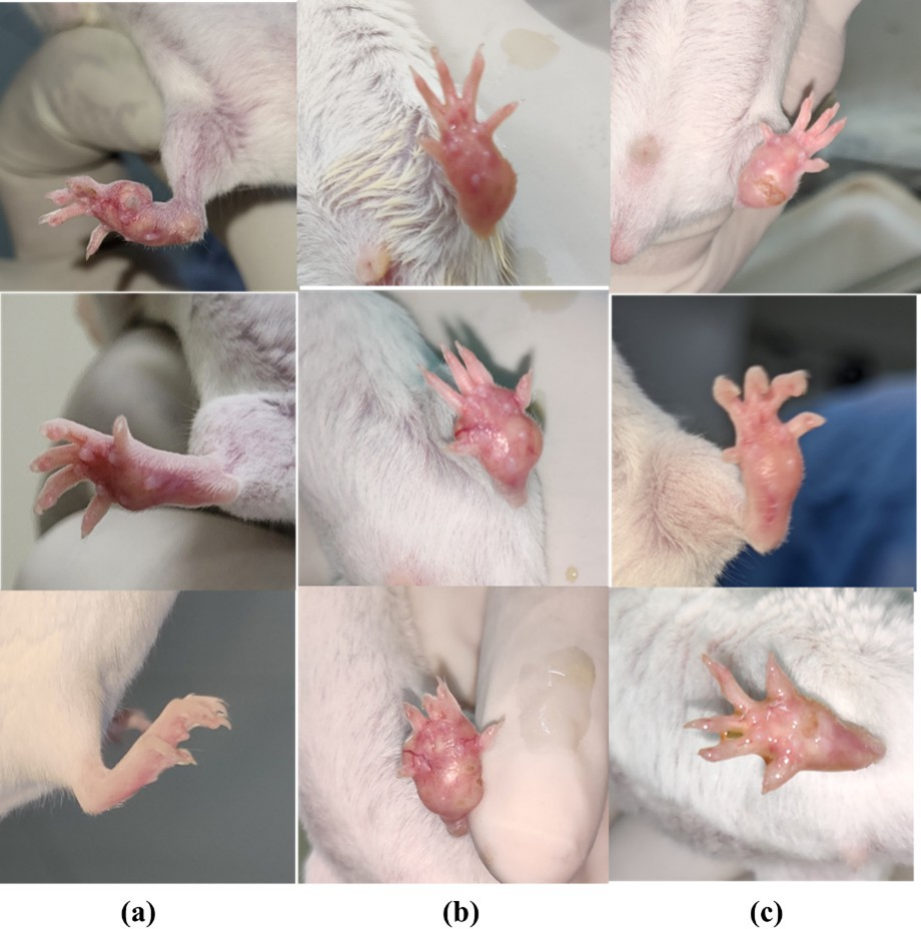
Combination Therapy **a.** Farnesol treated; **b.** Combination therapy; **c.** Paromomycin cream.

This parameter was conducted as a pilot study to confirm the *in vitro* findings in our previous study. As the findings from *in vitro* were antagonistic, hence, to confirm those results *in vivo*, groups were set for topical application only by combining the best dose of Farnesol and Paromomycin. The drugs reduced the lesions individually in line with the *in vitro* findings in previous, but the combination of both drugs capped each other’s parasite-killing effect and the infection increased even after treatment as shown in Fig 14B *in vivo*.

## 4. Discussion

Amongst leishmaniasis forms, CL is the most commonly encountered one. In India, CL outbreaks have mostly been documented in the arid areas of Rajasthan, Bikaner, and Gujarat, with scattered case reports from Punjab, Assam, and Haryana. However, recently there have been CL reports from other areas of the country, including Himachal Pradesh and Kerala [9–11]. A number of chemical, physical and surgical therapies have been recommended for the treatment of CL. However, drug resistance by parasite to chemotherapeutic agents remains a serious obstacle in the way of treating leishmaniasis [38]. Pentavalent antimonial therapy constitutes the first-line treatment for CL worldwide since 1945 such as Gentamycin and Miltefosine [18]. In a bid to escape disadvantages of chemotherapeutic agents including drug resistance, this study has been conducted to evaluate the *in vivo* effects of farnesol obtained from *L*. *major* in comparison with paromomycin. The necessity of the study was emphasized by the growing interest in natural products including medicinal plants as alternative therapies for CL. Several studies have already tackled the screening of plant extracts against leishmaniasis. Cutaneous leishmaniasis and visceral leishmaniasis clearly pose differing drug distributional challenges, with good dermal distribution through to more extensive tissue distribution requirements, respectively [39]. One of the novel treatment strategies that has received significant attention recently is the use of plant-based compounds. Farnesol, a sesquiterpene has found increasing biological and therapeutic applications [40]. Since, it is a self-secreted quorum-sensing molecule that has also been found to have antiviral, anticancer, and anti-protozoan properties. This study, however, clearly indicated high efficacy of farnesol in inhibiting promastigotes and amastigotes growth during *in vitro*, *in silico* investigations in previous studies and *in vivo* experimentations in the present study. Given the lack of data about the natural ingredients of these plants and their effects against leishmaniasis, this study aimed to investigate the therapeutic effects of farnesol on leishmanial lesions inflicted on BALB/c mice by *L*. *major* induced cutaneous leishmaniasis compared with Paromomycin effects and combination therapy. This is, to the best of our knowledge, the first study to explore the action of Farnesol on cutaneous leishmaniasis in BALB/c mouse model.

In our previous investigations, we found that farnesol exhibited promising anti-leishmanial activity both *in vitro* and *in silico*. It demonstrated dose-dependent susceptibility against L. major promastigotes and amastigotes, with IC50 and IC90 values approximately half of those observed for the FDA-approved drug paromomycin. Farnesol induced apoptosis in *L*. *major* promastigotes and its potential mechanism of action may involve inhibition of Lanosterol 14-demethylase, a key enzyme in the ergosterol production pathway.

The pharmacokinetic profile of Farnesol reveals a Log kp value of -3.81 cm/s, falling within the recommended range for topical drug application as determined by the Swiss-ADME profiling [41]. Furthermore, because of this drug’s high lipophilicity (Log *P*_o/w_ = 4.32), it is an indication of it passes through several membrane barriers and a significant pH barrier to reach the intracellular amastigotes in the phagolysosomes of most macrophages and stop their proliferation. It also follows all drug likeliness profiles according to ADMET profiles like Veber, Lipinski’s five rules, Ghose, Egan [41]. The bioavailability score was 0.55 which indicates that farnesol gets distributed evenly in the biological system, and suggests that it could be anticipated to produce excellent outcomes as a drug in an *in vivo* system.

The aforementioned findings provided a foundation for advancing towards in vivo investigations of Farnesol. For both the topical and oral groups, a range of three doses was selected from a broader range during the pilot study. Following inoculation of the mice hindfoot with the pathogen, lesion development occurred after 3–4 weeks, with subsequent increase in size and eruption within 1–2 weeks thereafter. The anti-leishmanial activity of farnesol was assessed through various parameters including measurement of lesion sizes, parasite load, changes in body weight, lymph node and spleen weight, and histological analysis. GC-MS spectra analysis of farnesol ointment revealed the absence of the hydroxyl group typically present in farnesol, indicating its presence in the ointments. Additionally, all samples containing farnesol exhibited peaks that aligned with the farnesol standard, confirming the presence of farnesol in each of the four ointment concentrations (Fig 2). Throughout the study, aside from a temperature increase felt by hand and lethargy at the peak of infection (i.e., two months after infection), no discernible changes in the mice’s weight were observed.

The lesion sizes in the test groups (treated with farnesol at 250 mM/ml) were significantly smaller than those in the control group, and paromomycin-treated group topically. There was a significant reduction in footpad thickness of farnesol-treated mice orally and topically at even lower concentrations than that of the standard drug (Fig 8). Vernier calliper was used to measure the footpad thickness on Days 1, 5, and 10 of the topical application treatment and Days 1, 10, and 20 of the oral treatment. Compared to Paromomycin at 50 mg/kg, mice treated with farnesol were completely healed, when given oral doses of 25 mg/kg and topical doses of 250 mM/ml (p > 0.001). The size and weight of popliteal lymph nodes of farnesol-treated groups were significantly reduced compared to Paromomycin and positive control groups orally and topically (p > 0.05), while spleen size and weight were not significantly different from the negative control group.

Combination therapy was carried out in continuation of previous *in vitro* studies. Like the *in vitro* findings, the combination of both drugs reduced their combined ability to fight off infection. While they displayed their normal killing effects individually.

Parasite load in the lymph node cells for treated groups were significantly very low when compared to positive controls for both the topical and oral groups of Farnesol. LD index for 500 mM/ml farnesol was 2.22 compared to paromomycin’s 13.13 ± 9.28 and was significantly very lower than the positive control of 82.77 ± 20.38 for mice given topical treatment. In oral treatment groups, the LD Index was 1.2 for 25mg/kg Farnesol and 78.34 ± 20 (p>0.05) for the positive control (Table 6). Comparatively speaking, there was a greater reduction in parasite load in mice groups that were given oral treatment (Table 7) as opposed to topical treatment, where the LD index for 500 mM was 2.22 in contrast to oral where the LD Index was 1.2 for 25mg/kg. Histiocytes /macrophages migrated to the lymph nodes of oral and topical groups of both paromomycin and Farnesol treated groups, and LD bodies were observed, according to histopathological analysis but also there was no granuloma formation in the treated groups as opposed to positive control which thereby, confirms some inhibitory mechanism of the drug towards the eradication of the parasites and control of disease in the lymphatic cells. There was no evidence of LD bodies or macrophages in histiocytes, indicating that the spleen was not infected. These findings point towards the probable lead that farnesol might be used as an alternative anti-leishmanial drug.

The lesions from cutaneous leishmaniasis cause abrasions of the epidermis and mucosa which might make a favourable environment for bacterial and fungal coinfections with increase the complications associated with the disease. We might be able to get a drug with broad-spectrum activity efficacious against cutaneous leishmaniasis and other coinfections associated with it, as various studies have reported farnesol’s efficacy on many pathogenic microbial species.

While the mechanism of farnesol inhibition of *Leishmania* parasites may involve the inhibition of Lanosterol-14 demethylase, further experimental analysis is necessary to provide more accurate information. *In silico* results can be complemented by protein tagging and targeting to confirm the mechanism of action. Additionally, extensive laboratory experiments are required to investigate any potential off-target effects and mechanisms of action of farnesol. Farnesol is a hydrophobic drug that presents challenges for oral delivery due to its limited solubility in water. However, these obstacles can be overcome by formulating the drug in an edible oil, such as castor oil, commonly used for the oral administration of lipid-soluble drugs. Farnesol has a bioavailability score of 0.55 (AMET), indicating its ability to effectively circulate and distribute throughout the body following oral administration.

## 5. Conclusions

In conclusion, farnesol demonstrated promising anti-leishmanial activity against cutaneous leishmaniasis (CL) in a BALB/c mouse model. *In vivo* experiments confirmed the effectiveness of farnesol in reducing lesion sizes and footpad thickness compared to controls. Lymph node size and weight were significantly decreased, while the spleen remained unaffected. Combination therapy did not enhance the drug’s effects. Histopathological analysis supported farnesol’s inhibitory mechanism in lymphatic cells. Farnesol also shows potential against coinfections associated with CL. Further investigations are needed to validate the mode of action, assess off-target effects, and optimize oral delivery. Overall, this study establishes farnesol as a promising alternative therapy for CL, warranting further research and development.

## Supporting information

S1 File All the crucial data important to the study, has already been included in the main manuscript. Gel/blot original images have been included in the supplementary files.
(PDF)

## References

[1] Cook G. C. and Zumla A., “History of tropical medicine, and medicine in the tropics,” Mansons Trop. Dis., vol. 22, pp. 1–8, 2008.

[2] Ready P. D., “Biology of phlebotomine sand flies as vectors of disease agents,” Annu. Rev. Entomol., vol. 58, pp. 227–250, 2013. doi: 10.1146/annurev-ento-120811-153557 23317043

[3] Rather S. et al., “Clinical and epidemiological study of cutaneous leishmaniasis in two tertiary care hospitals of Jammu and Kashmir: an emerging disease in North India,” Int. J. Infect. Dis., vol. 103, pp. 138–145, 2021. 10.1016/j.ijid.2020.11.002 33181331

[4] Okwor I. and Uzonna J., “Social and economic burden of human leishmaniasis,” Am. J. Trop. Med. Hyg., vol. 94, no. 3, p. 489, 2016. doi: 10.4269/ajtmh.15-0408 26787156 PMC4775878

[5] Alvar J. et al., “Leishmaniasis Worldwide and Global Estimates of Its Incidence,” PLOS ONE, vol. 7, no. 5, p. e35671, May 2012, doi: 10.1371/journal.pone.0035671 22693548 PMC3365071

[6] Zijlstra E. E., “PKDL and other dermal lesions in HIV co-infected patients with leishmaniasis: review of clinical presentation in relation to immune responses,” PLoS Negl. Trop. Dis., vol. 8, no. 11, p. e3258, 2014. doi: 10.1371/journal.pntd.0003258 25412435 PMC4238984

[7] Goossens A. and Merckx L., “Allergic contact dermatitis from farnesol in a deodorant,” Contact Dermatitis, vol. 37, no. 4, pp. 179–180, 1997. doi: 10.1111/j.1600-0536.1997.tb00192.x 9385513

[8] Reithinger R., Dujardin J.-C., Louzir H., Pirmez C., Alexander B., and Brooker S., “Cutaneous leishmaniasis,” Lancet Infect. Dis., vol. 7, no. 9, pp. 581–596, 2007. doi: 10.1016/S1473-3099(07)70209-8 17714672

[9] Kumar R., Bumb R. A., Ansari N. A., Mehta R. D., and Salotra P., “Cutaneous leishmaniasis caused by Leishmania tropica in Bikaner, India: parasite identification and characterization using molecular and immunologic tools,” Am. J. Trop. Med. Hyg., vol. 76, no. 5, pp. 896–901, 2007. 17488912

[10] Sharma R. C., Mahajan V. K., Sharma N. L., and Sharma A., “A new focus of cutaneous leishmaniasis in Himachal Pradesh (India),” Indian J Dermatol Venereol Leprol, vol. 69, no. 2, pp. 170–2, 2003. 17642870

[11] Simi S. M., Anish T. S., Jyothi R., Vijayakumar K., Philip R. R., and Paul N., “Searching for cutaneous leishmaniasis in tribals from Kerala, India,” J. Glob. Infect. Dis., vol. 2, no. 2, p. 95, 2010. doi: 10.4103/0974-777X.62874 20606960 PMC2889671

[12] Sharma N. L. et al., “Localized cutaneous leishmaniasis due to Leishmania donovani and Leishmania tropica: preliminary findings of the study of 161 new cases from a new endemic focus in himachal pradesh, India.,” Am. J. Trop. Med. Hyg., vol. 72, no. 6, pp. 819–824, 2005. 15964970

[13] Jara M. et al., “Real-time PCR assay for detection and quantification of Leishmania (Viannia) organisms in skin and mucosal lesions: exploratory study of parasite load and clinical parameters,” J. Clin. Microbiol., vol. 51, no. 6, pp. 1826–1833, 2013. doi: 10.1128/JCM.00208-13 23554201 PMC3716068

[14] Palatnik-de-Sousa C. B., “Vaccines for canine leishmaniasis,” Front. Immunol., vol. 3, p. 69, 2012. doi: 10.3389/fimmu.2012.00069 22566950 PMC3342354

[15] Sosa N. et al., “Topical paromomycin for New World cutaneous leishmaniasis,” PLoS Negl. Trop. Dis., vol. 13, no. 5, p. e0007253, 2019. doi: 10.1371/journal.pntd.0007253 31048871 PMC6497224

[16] Prabuseenivasan S., Jayakumar M., and Ignacimuthu S., “In vitro antibacterial activity of some plant essential oils,” BMC Complement. Altern. Med., vol. 6, no. 1, pp. 1–8, 2006. doi: 10.1186/1472-6882-6-39 17134518 PMC1693916

[17] Ramage G., Saville S. P., Wickes B. L., and López-Ribot J. L., “Inhibition of Candida albicans biofilm formation by farnesol, a quorum-sensing molecule,” Appl. Environ. Microbiol., vol. 68, no. 11, pp. 5459–5463, 2002. doi: 10.1128/AEM.68.11.5459-5463.2002 12406738 PMC129887

[18] Le Pape P., “Development of new antileishmanial drugs–current knowledge and future prospects,” J. Enzyme Inhib. Med. Chem., vol. 23, no. 5, pp. 708–718, 2008. doi: 10.1080/14756360802208137 18671165

[19] Ishizaka H., Yamada H., and Sasaki K., “Volatile compounds in the flowers of Cyclamen persicum, C. purpurascens and their hybrids,” Sci. Hortic., vol. 94, no. 1–2, pp. 125–135, 2002.

[20] Krupčík J., Gorovenko R., Špánik I., Sandra P., and Armstrong D. W., “Enantioselective comprehensive two‐dimensional gas chromatography. A route to elucidate the authenticity and origin of Rosa damascena Miller essential oils,” J. Sep. Sci., vol. 38, no. 19, pp. 3397–3403, 2015. doi: 10.1002/jssc.201500744 26235111

[21] Azanchi T., Shafaroodi H., and Asgarpanah J., “Anticonvulsant activity of Citrus aurantium blossom essential oil (neroli): involvment of the GABAergic system.,” Nat. Prod. Commun., vol. 9, no. 11, pp. 1615–1618, 2014. 25532295

[22] Rodrigues Goulart H., Kimura E. A., Peres V. J., Couto A. S., Aquino Duarte F. A., and Katzin A. M., “Terpenes arrest parasite development and inhibit biosynthesis of isoprenoids in Plasmodium falciparum,” Antimicrob. Agents Chemother., vol. 48, no. 7, pp. 2502–2509, 2004. doi: 10.1128/AAC.48.7.2502-2509.2004 15215101 PMC434199

[23] Dos Reis D. B. et al., “Synthesis and biological evaluation against Mycobacterium tuberculosis and Leishmania amazonensis of a series of diaminated terpenoids,” Biomed. Pharmacother., vol. 84, pp. 1739–1747, 2016. doi: 10.1016/j.biopha.2016.10.112 27876209

[24] Sharma H., Sehgal R., Shekhar N., Kaur U., and Medhi B., “Antiparasitic effect of Farnesol against Leishmania major: a rationale from in vitro and in silico investigations.” Preprints, Dec. 23, 2022. doi: 10.20944/preprints202212.0458.v1 PMC10627473 37930969

[25] Sousa A. Q., Frutuoso M. S., Moraes E. A., Pearson R. D., and Pompeu M. M., “High-dose oral fluconazole therapy effective for cutaneous leishmaniasis due to Leishmania (Vianna) braziliensis,” Clin. Infect. Dis., vol. 53, no. 7, pp. 693–695, 2011. doi: 10.1093/cid/cir496 21890773

[26] Derengowski L. S. et al., “Antimicrobial effect of farnesol, a Candida albicans quorum sensing molecule, on Paracoccidioides brasiliensis growth and morphogenesis,” Ann. Clin. Microbiol. Antimicrob., vol. 8, no. 1, pp. 1–9, 2009. doi: 10.1186/1476-0711-8-13 19402910 PMC2681445

[27] Wijnant G.-J., Van Bocxlaer K., Yardley V., Murdan S., and Croft S. L., “Efficacy of paromomycin-chloroquine combination therapy in experimental cutaneous leishmaniasis,” Antimicrob. Agents Chemother., vol. 61, no. 8, pp. e00358–17, 2017. doi: 10.1128/AAC.00358-17 28607026 PMC5527568

[28] Venuprasad K. et al., “Human neutrophil-expressed CD28 interacts with macrophage B7 to induce phosphatidylinositol 3-kinase-dependent IFN-γ secretion and restriction of Leishmania growth,” J. Immunol., vol. 169, no. 2, pp. 920–928, 2002. 12097397 10.4049/jimmunol.169.2.920

[29] Gupta N., Goyal N., and Rastogi A. K., “In vitro cultivation and characterization of axenic amastigotes of Leishmania,” Trends Parasitol., vol. 17, no. 3, pp. 150–153, 2001. doi: 10.1016/s1471-4922(00)01811-0 11286801

[30] Yadav M., Gupta I., and Malla N., “Kinetics of immunoglobulin G, M, A and IgG subclass responses in experimental intravaginal trichomoniasis: prominence of IgG1 response,” Parasite Immunol., vol. 27, no. 12, pp. 461–467, 2005. doi: 10.1111/j.1365-3024.2005.00800.x 16255745

[31] Kotmire S., Gupta I., Ganguly N. K., and Koicha M., “Study of T-lymphocyte subpopulation in HBsAg-positive pregnant women.,” Acta Virol., vol. 37, no. 6, pp. 459–465, 1993. 8010184

[32] Ibrahim K. E., Al-Mutary M. G., Bakhiet A. O., and Khan H. A., “Histopathology of the liver, kidney, and spleen of mice exposed to gold nanoparticles,” Molecules, vol. 23, no. 8, p. 1848, 2018. doi: 10.3390/molecules23081848 30044410 PMC6222535

[33] Dey R. et al., “Characterization of cross-protection by genetically modified live-attenuated Leishmania donovani parasites against Leishmania mexicana,” J. Immunol., vol. 193, no. 7, pp. 3513–3527, 2014. doi: 10.4049/jimmunol.1303145 25156362 PMC6480318

[34] Lee J., Lee Y., Ha J., Yoo M., and Jang H. W., “Simultaneous determination of four bioactive compounds in Korean rice wine (makgeolli) by solvent extraction coupled with gas chromatography-mass spectrometry,” Int. J. Food Prop., vol. 21, no. 1, pp. 139–146, 2018.

[35] Smith D. F., Searle S., Ready P. D., Gramiccia M., and Ben-Ismail R., “A kinetoplast DNA probe diagnostic for Leishmania major: sequence homologies between regions of Leishmania minicircles,” Mol. Biochem. Parasitol., vol. 37, no. 2, pp. 213–223, 1989. doi: 10.1016/0166-6851(89)90153-9 2558320

[36] Dey S. et al., “Combination of Mycobacterium indicus pranii and heat-induced promastigotes cures drug-resistant Leishmania infection: critical role of interleukin-6-producing classical dendritic cells,” Infect. Immun., vol. 88, no. 6, pp. e00222–19, 2020. doi: 10.1128/IAI.00222-19 32229617 PMC7240079

[37] Mukherjee D. et al., “Targeting the trypanothione reductase of tissue-residing Leishmania in hosts’ reticuloendothelial system: A flexible water-soluble ferrocenylquinoline-based preclinical drug candidate,” J. Med. Chem., vol. 63, no. 24, pp. 15621–15638, 2020. doi: 10.1021/acs.jmedchem.0c00690 33296601

[38] Maleki F., Zarebavani M., Mohebali M., Dayer M. S., Hajialiani F., and Tabatabaie F., “In vitro and in vivo susceptibility of Leishmania major to some medicinal plants,” Asian Pac. J. Trop. Biomed., vol. 7, no. 1, pp. 37–42, 2017.

[39] De Rycker M., Wyllie S., Horn D., Read K. D., and Gilbert I. H., “Anti-trypanosomatid drug discovery: progress and challenges,” Nat. Rev. Microbiol., pp. 1–16, 2022. 10.1038/s41579-022-00777-y PMC9395782 35995950

[40] Yoo S., Murata R. M., and Duarte S., “Antimicrobial traits of tea-and cranberry-derived polyphenols against Streptococcus mutans,” Caries Res., vol. 45, no. 4, pp. 327–335, 2011. doi: 10.1159/000329181 21720161 PMC3130978

[41] Yang R., Xia Y., Xian J., Yu H., Yan B., and Cheng B., “Identification of Potential Dual Farnesol X Receptor/Retinoid X Receptor α Agonists Based on Machine Learning Models, ADMET Prediction and Molecular Docking,” ChemistrySelect, vol. 7, no. 28, p. e202200715, 2022.

